# Nurse Practitioner–Pharmacist Collaboration for Medication Regimen Simplification: Pilot and Feasibility Study of a New Telehealth Model of Care

**DOI:** 10.1155/jonm/1321189

**Published:** 2026-08-29

**Authors:** Choon Ean Ooi, Angelita Martini, Rebecca Walton, Brigid McInerney, Lakkhina Troeung, J. Simon Bell

**Affiliations:** ^1^ Centre for Medicine Use and Safety, Faculty of Pharmacy and Pharmaceutical Sciences, Monash University, Parkville, Victoria, Australia, monash.ac.za; ^2^ Calvary Health Care, Sydney, Australia; ^3^ The University of Notre Dame Australia, Institute for Health Research, Fremantle, Western Australia, Australia, nd.edu.au; ^4^ Brightwater Research Centre, Brightwater Care Group, Inglewood, Western Australia, Australia; ^5^ Pharmacy Department, Monash Health, Clayton, Victoria, Australia, monashhealth.org; ^6^ Duke-NUS Medical School, Singapore, duke-nus.edu.sg

**Keywords:** aged care, collaborative care, medication management, medication simplification, nurse practitioners, pharmacists

## Abstract

**Background:**

Telehealth can increase access to medical and allied health services in residential aged care facilities (RACFs).

**Objectives:**

This study investigated the feasibility of a telehealth collaborative care model involving on‐site nurse practitioner and off‐site clinical pharmacist to simplify complex medication regimens in RACFs.

**Methods:**

This was a pragmatic, nonrandomized pilot and feasibility study in three Western Australian RACFs. A nurse practitioner and an off‐site clinical pharmacist applied a validated five‐step regimen simplification tool. The nurse practitioner identified residents who may benefit and had a desire to simplify their regimen. The nurse practitioner and pharmacist then conducted video case conferences to discuss simplification opportunities. The nurse practitioner decided which simplification opportunities to implement in consultation with residents and general medical practitioners (GPs). The primary outcome was feasibility (recruitment and retention, protocol adherence, and stakeholder acceptability). Secondary outcomes included change in the number of medication administration times per day, medication and behavioral incidents, falls and fractures, and hospitalization at 4 months.

**Results:**

Telehealth collaborative care was delivered according to the protocol, with one or more simplification opportunities identified for 18 of 21 residents. The most frequent simplification opportunities involved changing a dose time or formulation. The intervention was well received by the interviewed stakeholders. At the 4‐month follow‐up, 47% of residents had at least one simplification recommendation implemented and one‐third had a reduced number of regular daily medication administration times. Compared to the previous quarter, there was a non‐significant decline in medication incidents (24%–18% of residents), behavioral incidents (48%–35%), and falls and fractures (48%–41%). No hospitalizations were reported among residents who completed follow‐up.

**Conclusions:**

This nurse practitioner–pharmacist telehealth intervention was feasible and acceptable. Further adequately powered trials to detect clinical outcomes are warranted.

**Implications for Nursing Management:**

Nursing managers should actively support telehealth‐enabled interdisciplinary collaboration that promotes nurse practitioner leadership and fosters partnerships with pharmacists and GPs to advance medication regimen simplification and optimize medication management processes. Such initiatives may enhance the efficiency of medication administration, improve care coordination, and strengthen medication safety within aged care environments.

## 1. Introduction

There is a growing proportion of people living in residential aged care facilities (RACFs) with complex medication regimens. Up to 63% of Australian residents use nine or more regular medications [1], and more than one‐third require five or more administration times per day [2]. Regular medications are medications that are administered on a regular basis or consistent schedule. Complex medication regimens have been associated with adverse drug events [3], medication nonadherence [3], medication self‐administration errors [4], hospitalization [1, 3], hospital re‐admission [3], hospital discharge to non‐home settings [5], and mortality [6]. Complexity can arise due to multiple medications use, multiple medication administration times, use of non‐oral formulations, specific preparation, or administration requirements [7]. Complex medication regimens can be burdensome for residents, particularly for residents with dementia, and are costly for aged care providers in terms of nursing time [8]. Polypharmacy has been a component of Australia’s National Aged Care Mandatory Quality Indicator Program [9] since July 2021. The portion of residents with more than four daily medication administration times is a voluntary quality indicator in public‐sector RACFs in the state of Victoria [10].

Medication regimen simplification involves strategies such as consolidating dosing times, using long‐acting in preference to short‐acting formulations, standardizing routes of administration, and switching from multiple single‐ingredient to combination products [11]. A cluster randomized controlled trial in South Australian RACFs demonstrated that one‐off application of a structured and validated regimen simplification tool (The Medication Regimen Simplification Guide for Residential Aged CarE, MRS GRACE) achieved a sustained reduction in residents’ medication regimen complexity [12, 13].

There is a recognized need for new multidisciplinary models of care for older people with complex care needs [14]. Pharmacists [15] and nurse practitioners [16] have increasingly important roles in medication management in RACFs. Although pharmacists have been funded to provide medication review services in Australian RACFs since 1997, most RACFs do not have an on‐site pharmacist, highlighting the need for innovative collaborative approaches to medication management. To our knowledge, no previous studies have evaluated models in which nurse practitioners and pharmacists collaborate to improve medication management in Australian RACFs.

Telehealth services have become more widely adopted since the 2019 coronavirus (COVID‐19) pandemic [17]. Many recent studies have examined the provision of care by nurse practitioners via telehealth, primarily focusing on patient education [18]. A scoping review found that telehealth could also facilitate multidisciplinary collaboration among remote healthcare providers [19]. There has also been an increasing interest in telehealth for delivering medication management [20]. Telehealth could be utilized for provision of pharmacist services to RACFs that do not directly employ on‐site pharmacists. This could include RACFs in rural and remote areas that have less access to specialist medication management services. This study aimed to investigate the feasibility of telehealth collaborative care involving an on‐site nurse practitioner and an off‐site clinical pharmacist to simplify complex medication regimens in RACFs.

## 2. Methods

### 2.1. Design, Setting, and Participants

This was a non‐randomized pilot and feasibility study conducted at three RACFs operated by an independent, not‐for‐profit aged care organization in Western Australia. The three RACFs were in metropolitan Perth. Three RACFs, rather than the planned four, participated due to COVID‐19 restrictions. In Australia, RACFs are similar to nursing homes or long‐term care facilities in other countries and provide supported accommodation for people who are unable to continue living independently in their own homes [21]. The three RACFs employed a nurse practitioner as a member of staff. These nurse practitioners were also members of the aged care organization’s multidisciplinary Medication Advisory Committee (MAC) [22].

Resident participants were purposively recruited to include a diverse sample of residents with and without dementia and with varying levels of frailty. Residents who were non‐English‐speaking, had fewer than two daily medication administration times, were estimated to have less than three months to live, were deemed to be medically unstable, or had a public guardian were excluded. In Australia, public guardian is a public official or an agency appointed by a court or tribunal to make healthcare and lifestyle decisions on behalf of a person who no longer has decision‐making capacity. The full study protocol has been described previously [23].

### 2.2. Resident Recruitment

The nurse practitioner and a research officer employed by the aged care organization identified eligible residents with complex medication regimens at each RACF using predefined criteria. The nurse practitioner and research officer then consulted with the residential services manager (RSM) and the general medical practitioner (GP) of each eligible resident to determine whether the resident had the capacity to provide informed consent. When an eligible resident lacked the capacity to provide informed consent, the GP would complete the Guardianship and Administration Act 1990 Medical Research Decision Form in accordance with guidance from the Western Australian Department of Health [24] if they believed that the resident would benefit from participating in the study. Third‐party consent was then sought from the resident’s guardian, next of kin, or significant other if the GP determined that the resident was unable to make a reasonable judgment.

### 2.3. Intervention

The intervention involved an on‐site nurse practitioner working in partnership with an off‐site clinical pharmacist to apply the five‐step MRS GRACE tool (Box 1) for each participating resident [25].

**Box 1: Medication Regimen Simplification Guide for Residential Aged CarE (MRS GRACE) [25]**
 Consideration can be given to administering all medications at the same time each day unless the following apply:
1. Is there a resident‐related factor that precludes simplification?
2. Is there a regulatory or safety imperative that precludes simplification?
3. Is simplification likely to result in any clinically significant drug–drug, drug–food, or drug–time interactions?
4. Is there no alternative formulation available that can support less complex dosing?
5. Is simplification likely to result in any unintended consequences for the resident or facility?
 Reproduced from Chen EY, Sluggett JK, Ilomäki J, et al. Development and validation of the Medication Regimen Simplification Guide for Residential Aged CarE (MRS GRACE). Clin Interv Aging. 2018 May 18; 13:975‐986. doi: 10.2147/CIA.S158417. Further details on each simplification criterion are available in Chen et al. [25].

**FIGURE 1:**
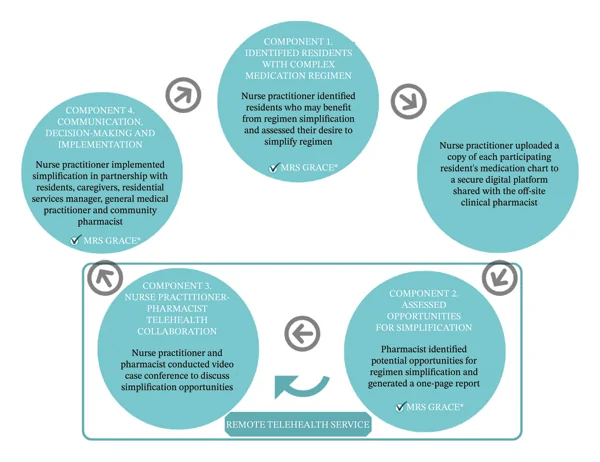
Nurse practitioner–pharmacist telehealth collaboration to simplify complex medication regimens in residential aged care facilities [23].^∗^MRS GRACE is a five‐step, implicit structured tool to identify opportunities for simplifying medication regimens [25] and was applied by the nurse practitioner or off‐site clinical pharmacist in components 1, 2, and 4 of the study intervention phase.

The intervention had four main components (Figure 1) [23]. The nurse practitioner assessed each participating resident’s desire to simplify their medication regimen and any regulatory or safety imperatives that could preclude simplification (MRS GRACE steps 1 and 2). The nurse practitioner provided a digital copy of each participating resident’s medication chart to the off‐site clinical pharmacist. The pharmacist, using their expertise in pharmaceutical products, formulations, and interactions, identified opportunities for regimen simplification (MRS GRACE steps 3 and 4). A structured one‐page report outlining potential opportunities for regimen simplification and other comments, such as suggested monitoring following implementation of simplification recommendations, where appropriate, was prepared by the pharmacist for the nurse practitioner and GP. The pharmacist then discussed the report for each resident with the nurse practitioner via a video case conference. The nurse practitioner then discussed potential regimen simplification opportunities with the resident and their caregiver, RSM, GP, and community pharmacist (MRS GRACE step 5). The nurse practitioner or GP, in consultation with the resident and their caregiver, made the decision on whether to implement any simplification opportunities. The implementation process was guided by a protocol adapted from the SIMPLER study [8] and endorsed by the aged care organization’s MAC. Any simplification that altered medication administration times required approval and implementation by the nurse practitioner or GP, whereas changes to a prescription issued by a GP (e.g. new medication strength or formulation) required the GP’s approval before implementation.

### 2.4. Usual Care

Participating residents continued to receive all usual clinical and care services throughout the study period, including the residential medication management reviews (RMMRs) provided by GPs and accredited pharmacists [26].

### 2.5. Data Collection

Data were collected at baseline and 4‐month follow‐up by an independent research officer. Demographic, medication, and other clinical data were extracted from each resident’s electronic medical record and medication chart.

### 2.6. Primary and Secondary Outcomes

The primary outcome was study feasibility, comprising recruitment and retention, implementation of simplification opportunities, protocol adherence, and stakeholder acceptability of intervention delivery. Feasibility was assessed using a mixed‐methods approach that included document analysis (recruitment logs, field notes, and the pharmacist’s reports) and semistructured interviews. Due to the challenges in arranging focus group with the participants, semistructured interviews were chosen as a more practical alternative. The interview questions were adapted from the SIMPLER study [27]. The interviews were audiotaped, transcribed verbatim, and analyzed using relevant domains of Grant et al.’s framework for process evaluation of cluster randomized controlled trials of health interventions [28]. Qualitative methods were chosen to explore and understand the range of different stakeholder insights on the new intervention introduced.

Secondary outcomes included the change in the number of medication administration times over a 24‐h period for regular medications which was calculated based on the number of regular daily medication administration times at baseline and 4‐month follow‐up. Short‐term medications, nurse‐initiated medications, *pro-re-nata* (PRN) medications, and nutritional drinks were excluded when calculating the number of administration times. Other secondary outcomes were medication incidents, behavioral incidents, falls and fractures, and hospitalization during the 4‐month pre‐ and 4‐month post‐regimen simplification. The number and types of medication simplification opportunities identified by the pharmacist, as well as the time spent by the pharmacist, were also assessed. Further details regarding the collection and analyses of primary and secondary outcome data have been provided in the published study protocol [23].

### 2.7. Statistical Analyses

Formal sample size calculations were not considered necessary for this pilot and feasibility study. The proposed sample size (*n* = 30) aligned with a previous Australian pilot and feasibility study on medication regimen simplification for home care packages recipients [29]. Quantitative data were analyzed using Microsoft Excel.

### 2.8. Ethics Approval

Ethical approval was obtained from the Monash University Human Research Ethics Committee (ID: 30171). The study procedures were endorsed by the aged care organization’s MAC. Written informed consent was obtained from all participants with decision‐making capacity. For eligible residents who lacked the capacity to provide informed consent, their GP made the required determination through the third‐party consent process in accordance with Western Australian Department of Health guidance. All participant information was kept confidential through de‐identification and secure data management procedures.

## 3. Results

### 3.1. Participant Characteristics

Twenty‐one residents consented to participate between September 2022 and February 2023 (Table 1). All residents who consented to participate received the intervention. Semistructured interviews were conducted in June 2023 with five participants (research officer, nurse practitioner, pharmacist, GP, and caregiver of a participating resident).

**TABLE 1:** Baseline characteristics of participating residents.

Characteristics	Participants, *n* = 21
Age, years (mean ± SD)	83.1 ± 9.6
Female (*n*, %)	15 (71.4%)
Medication use (mean ± SD)	
Total number of medications charted^a^	15.1 ± 5.1
Number of medications charted for regular use^b^	12.8 ± 4.2
Number of administration times for medications taken regularly^b^ (*n*, %)	
Twice daily	1 (4.8%)
Three times daily	4 (19.0%)
Four times daily	11 (52.4%)
Five times daily	5 (23.8%)

^a^Included regular and PRN medications; and excluded nutritional drinks. None of the participants was charted for short‐term or nurse‐initiated medications.

^b^Excluded PRN medications and nutritional drinks. None of the participants was charted for short‐term or nurse‐initiated medications.

### 3.2. Primary Outcome: Study Feasibility

#### 3.2.1. Recruitment and Retention

**FIGURE 2:**
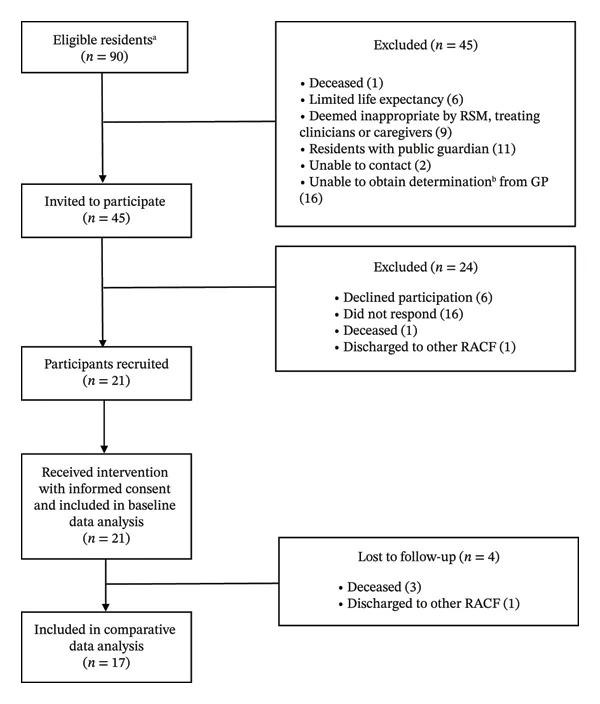
Participant flowchart. ^a^Permanent residents who were English‐speaking and with two or more daily medication administration times. ^b^When an eligible resident did not have the capacity to consent, their GP then made a determination (i.e., by completing the Guardianship and Administration Act 1990 (GAA) medical research decision form for the resident) according to the guidance from the Western Australian Department of Health [24]. RSM, residential services manager; GP, general medical practitioner; RACF, residential aged care facilities.

Of 90 eligible residents at the three RACFs, 45 met the inclusion criteria and were invited to participate (Figure 2). Of these, 21 (47%) agreed to participate through third‐party consent (spouse, *n* = 7; son, 7; daughter, 6; daughter‐in‐law, 1). The guardian, next of kin, or significant other declined participation for six of 45 residents (13%). Reasons for declining included resistance to changes in the resident’s medications or the unavailability of third‐party consent. Four participating residents (deceased, *n* = 3; discharged to other RACF, 1) were lost to follow‐up at 4‐month.

The target sample size of 30 residents was revised downward due to the inclusion of three rather than four RACFs. There were also recruitment challenges, including the resident’s GP not being available to complete the Guardianship and Administration Act 1990 Medical Research Decision Form (18%) and the resident’s guardian, next of kin, or significant other not responding to the study invitation (18%). Other key barriers and enablers to recruitment were identified through the interviews.

“… COVID‐19 was also a big barrier. We were trying to recruit right when WA was experiencing its first proper COVID‐19 wave, and there were extremely strict measures about entering sites, which limited my ability to contact residents who could consent themselves… And as each site has a different service manager – you have varying degrees of interest, time and engagement.” [Research officer]

It was suggested that providing families and/or residents with a face‐to‐face information session could help them better understand research involvement.

#### 3.2.2. Protocol Adherence

The quantitative document analysis and qualitative interviews (pharmacist, nurse practitioner and GP) suggested the intervention was delivered according to the protocol.

The nurse practitioner identified no resident‐related factors or regulatory/safety imperatives that precluded regimen simplification. The pharmacist spent an average of 16 minutes assessing each resident’s medication chart and 10 minutes preparing each report. Of the 21 participating residents, 18 (86%) had at least one simplification opportunity identified. The most common simplification opportunities involved changing a medication dose time or formulation (Table 2).

**TABLE 2:** Simplification opportunities identified by the pharmacist.

Number of simplification opportunities identified	Participants, *n* = 21
None	3 (14.3%)
One	10 (47.6%)
Two	5 (23.8%)
Three	3 (14.3%)

**Types of simplification opportunities identified**	**Simplification opportunities identified, n = 29**

Change of dose time^a^	13 (44.8%)
Change of formulation^b^	10 (34.5%)
Change of dosing frequency^c^	5 (17.2%)
Other^d^	1 (3.5%)

^a^Consolidation of dosing times (e.g., move medications given at 1300–1200 and 1500–1900 so they can be taken with other regular medications).

^b^Switch from short‐acting to long‐acting formulations or from multiple single‐ingredient to combination products (e.g., change paracetamol 500 mg to paracetamol MR 665 mg, or combine latanoprost eye drops and timolol eye drops to latanoprost/timolol eye drops).

^c^Consolidation of dosing frequency (e.g., change cholecalciferol 1000 units 1 capsule twice daily to 2 capsules once daily).

^d^Cease duplicate medication order (e.g., cease duplicate order of diclofenac gel for a resident).

The nurse practitioner and pharmacist held video case conferences using the Zoom platform for all 21 residents across two sessions. Each case conference lasted an average of five minutes per resident with simplification opportunities. Simplification was not enacted for eight (44%) of the 18 residents with simplification opportunities identified because the nurse practitioner, in consultation with the families and GP, deemed that simplification may not be desirable. This was due to the resident’s preference or financial considerations.

Both the pharmacist and nurse practitioner had positive experiences in using MRS GRACE tool during intervention delivery. The pharmacist felt that MRS GRACE would be particularly useful for facilitating simplification by less experienced pharmacists.

“It’s similar I guess in terms of the way you approach a medication review in terms of trying to be pragmatic, trying to recommend things that ultimately benefit the resident or the patient but it was helpful and, what’s the word, not rewarding but it was a positive experience to I guess have the MRS GRACE workflow and have a real structure to back you up.” [Pharmacist]

“MRS GRACE is great… and it has changed how I actually look at medications overall as well. So, I think having that tool in place has been a huge benefit, and I think in future, going forward as well, it’s something that you take on and move forward with, having to be able to both look at and compact and simplify that whole medication regimen.” [Nurse practitioner]

The pharmacist noted a tendency toward conducting more comprehensive clinical reviews when assessing each resident’s medication charts for simplification opportunities.

“… it was essentially going top to bottom with the chart… a broader review in the sense that I think as a healthcare professional you’re going to be conscious of or looking for red flags beyond maybe what you’re being instructed to do… strange regimens or duplicate benzos, things like that, which perhaps not strictly part of the MRS GRACE workflow or what I was instructed to do but I felt necessary to bring up…” [Pharmacist]

The video case conferences facilitated a productive discussion of simplification opportunities and their suitability for implementation.

“… the liaison with the pharmacy was one of the biggest benefits. While I’m quite confident in my prescribing and looking at things, it was having that second person having a look at things, and picking up things that I would have thought that I had seen, but she (pharmacist) picked up things I hadn’t sort of picked up on. So I think the most beneficial part was that interaction with the pharmacy, definitely.” [Nurse practitioner]

#### 3.2.3. Stakeholder Acceptability of Intervention Delivery

The intervention was perceived by the interviewed stakeholders as beneficial for the aged care organization and its residents, as it reduced the burden of medication administration and had the potential to minimize medication administration errors.

“I think what’s more valuable is the like ease of delivery and ease of monitoring them and the ease towards the staff.” [GP]

“I guess the thing with the organization wise is that it gives to extra time for staff, having not to worry about medication, having to find the time, and also the risk of missing medications at different times throughout the day, if you have to give somebody medications QID, and we can manage to get that down to BD, the chances of missing those medications are far less. So the risk to the resident then reduces.” [Nurse practitioner]

The nurse practitioner commented on how well the intervention was received by the nursing staff at RACF sites. The GP believed the medication simplification review was complementary to clinically focused RMMR service.

“… the external pharmacist is probably look at more how to simplify them rather than rationalize them. So there is a bit of difference (to RMMR) … Simplification probably take off a fair bit of the pressure off the nurse’s workload and on top of that there are so many residents who don’t want to take three or four times a day medications anyway.” [GP]

The majority of the interviewees agreed on the increasing uptake, comfort, and familiarity with telehealth since the COVID‐19 pandemic. The nurse practitioner and pharmacist perceived that telehealth discussions were similar to face‐to‐face discussions for the purpose of exchanging pharmacological information about simplification opportunities. The pharmacist, although stating a preference for face‐to‐face interactions where possible, commented that the use of telehealth could facilitate RACF accessing medication management services provided by a pharmacist, especially for remote or regional areas. The GP also supported the use of telehealth in collaborative care models involving off‐site health care professionals.

“Telehealth works well because as far as the nurse practitioner knows the patient and the GPs, as GPs we know the patients anyway, and regardless of the pharmacist where they are, what they need is the information.” [GP]

At 4‐month follow‐up, 7 out of 15 participating residents with simplification opportunities identified had at least one simplification opportunity implemented. The majority were related to the change of dose time, followed equally by change of dose frequency, formulation, and revision of duplicate order.

The nurse practitioner reported that the intervention led to other nurses at RACFs paying more attention to selecting appropriate medication administration times when new medications were prescribed.

“… the staff will look at times of medications, rather than just having medications put in there. So they’ve actually thought about what times they’re going to give the medications, or what times that they feel those medications could be given, realistically, tying in with the different types of medications that they’re looking at. So I think it’s got them also thinking about simplifying their med rounds, themselves.” [Nurse practitioner]

Interviewees agreed there was potential for wider implementation of MRS GRACE. The GP welcomed the telehealth‐based collaborative care model studied but also expressed concern that some GPs might be territorial and uncomfortable working collaboratively. Building strong relationships between health and care team and fostering trust could facilitate collaboration. Funding and resources (staffing and equipment for telehealth) were the other barriers identified.

“… in addition to RMMR if you do have another sort of service provider available, I don’t know who’s going to fund it, but as far as is available somehow that will be valuable because we still struggle with the polypharmacy in aged care. So that will help us quite a bit to offload that burden.” [GP]

### 3.3. Secondary Outcomes

One‐third of the participating residents with simplification opportunities identified had a reduced number of regular daily medication administration times at 4 months (from five times daily to four times daily, *n* = 3; from four times daily to twice daily, *n* = 2) (Table 3).

**TABLE 3:** Change in secondary outcomes from baseline to 4‐month follow‐up.

Outcome	Baseline, T0	4‐month, T4
Number of residents with data, *n*	21	17^a^
Medication incidents		
Residents with incident, *n* (%)	5 (23.8)	3 (17.6)
Number of incidents, *n*	6	3
Mean number of incidents per resident (SD)^b^	1.2 (0.4)	1 (0.0)
Behavioral incidents		
Residents with incident, *n* (%)	10 (47.6)	6 (35.3)
Number of incidents, *n*	23	11
Mean number of incidents per resident (SD)^b^	2.3 (1.3)	1.8 (0.7)
Falls and fractures		
Residents with incident, *n* (%)	10 (47.6)	7 (41.2)
Number of incidents, *n*	31	38
Mean number of incidents per resident (SD)^b^	3.1 (2.6)	5.4 (5.3)
Hospitalizations		
Residents with incident, *n* (%)	2 (9.5)	0 (0.0)
Number of incidents, n	2	0
Mean number of incidents per resident (SD)^b^	1 (0.0)	0
Deceased, *n* (%)	—	3 (17.6)

Abbreviation: SD, standard deviation.

^a^Four participants were lost to follow up at 4‐month, T4.

^b^Calculated as number of incidents/number of residents with incident.

The proportion of residents with medication incidents, behavioral incidents, falls and fractures, and hospitalization was lower in the 4‐month period post‐simplification than in the 4‐month period pre‐simplification, but the differences were not statistically significant.

The mean number of medication and behavioral incidents per resident were similar from baseline to 4 months. There was an increase in the mean number of falls from 3.1 falls per resident at baseline to 5.4 falls per resident at 4 months (driven by an increase in falls in two residents).

None of the incidents (medication, behavioral, and falls) reported at 4 months were deemed related to medication regimen simplification. No participating residents who completed the follow‐up were hospitalized during the 4 months post‐simplification.

## 4. Discussion

This study demonstrated the feasibility of an innovative model of collaboration involving RACF nurse practitioners and off‐site clinical pharmacists. To our knowledge, this is the first Australian telehealth medication management service involving collaboration between a nurse practitioner and pharmacist. The intervention was well accepted by the interviewed stakeholders. Reducing the burden of medication administration was perceived as particularly beneficial for residents with cognitive impairment and helpful in relieving the nursing workload [27]. This may be valuable for aged care organizations seeking to attract and retain their workforce.

Resident recruitment was lower than originally anticipated, but most challenges were related to the COVID‐19 pandemic and obtaining third‐party consent, rather than the nature of the intervention itself. There were limited opportunities for the research officer, who recruited participants, to have face‐to‐face engagement with the residents and their guardians, next of kin, or significant others due to COVID‐19 restrictions. Not all eligible residents had the capacity to provide informed consent, and this necessitated third‐party consent. Despite these challenges, the recruitment rate of 47% was higher than a German study of pharmacist medication management services for RACF residents (34%) [30], the SIMPLER study in eight South Australian RACFs (38%) [27], and in another Australian study of regimen simplification for recipients of home care packages (12%) [31].

Our model was novel in that it involved prescribing nurse practitioners in residential aged care. However, other models of nurse‐pharmacist collaboration to improve medication safety have been reported in the community [32], acute care setting [33], and during transition of care [34]. Lack of role clarity, ineffective workflow, inadequate exchange of information, lack of remuneration, suboptimal use of technology, and suboptimal communication channels were reported in these previous models [32, 35]. This study implemented a range of measures to overcome some of these challenges. For example, to overcome workflow issues, the intervention delivery was guided by MRS GRACE tool with a stepwise approach for medication simplification. Similarly, to overcome issues related to lack of role clarity, the roles and responsibilities of the nurse practitioner and pharmacist were outlined in the study protocol [23]. Multidisciplinary collaboration was a feature of our model, and this may be important because different healthcare professionals may identify different regimen simplification opportunities [36].

In Australia, telehealth services are almost synonymous with video conferencing consultations and were initially used to support regional health professionals [37]. Telehealth has gained widespread popularity for healthcare delivery over the past 4 years. While COVID‐19 was a barrier to in‐person participant recruitment, the model investigated in this study is potentially well suited to ensuring provision of pharmacy services when physical distancing measures and other restrictions are in place. The familiarity with technology could have facilitated the intervention delivery in this pilot study. The nurse practitioner, pharmacist, and GP commented that telehealth was a viable alternative to face‐to‐face when there is adequate equipment, staffing, and funding for service delivery. The Australian Government permitted pharmacists to conduct RMMRs via telehealth during the COVID‐19 pandemic [38]. This study suggests that there may be value in extending this opportunity to a wider range of pharmacy services and in non‐pandemic times.

Telehealth‐based collaboration between RACF nurse practitioner and off‐site pharmacist looks promising and suitable for larger‐scale adoption and implementation. This could be especially true for RACFs in rural and remote areas where access to clinical pharmacy services is limited. For example, residents outside major cities are less likely to receive RMMRs within 90 days of admission than residents in Australian cities [39]. The Australian Government has announced funding for embedded on‐site pharmacists to work at aged care facilities [40]. Medication regimen simplification is one role that may be undertaken by on‐site pharmacists. While it is not envisaged that telehealth will replace the need for on‐site pharmacists, new models that provide flexibility in the implementation of pharmacy services may increase access across RACFs when there are skilled workforce shortages. Telehealth may also facilitate implementation and follow‐up of pharmacy services by on‐site pharmacists who work across different RACFs operated by the same aged care organization.

This was a pilot and feasibility study not powered to detect possible changes in the secondary outcomes of medication incidents, behavioral incidents, or falls and fractures. However, it was encouraging that there were non‐significant reductions in the proportion of residents who experienced these outcomes at the 4‐month follow‐up. Larger adequately powered studies are needed to investigate the possible impact of simplification on these outcomes. The 12‐month number of medication incidents almost halved in the intervention arm of SIMPLER trial (adjusted incidence rate ratio 0.56; 95% confidence interval 0.38–0.80), although this reduction was not significant when compared to a corresponding reduction in incidents in the control group [41].

### 4.1. Strengths and Limitations

The study investigated a new workforce model for simplifying complex medication regimens. The intervention involved a high level of stakeholder engagement in both its design and delivery. There were several study limitations. This study was conducted within RACFs operated by a single aged care organization which employed nurse practitioners and involved one off‐site clinical pharmacist. There is a possibility of selection bias, as eligible residents were identified by a nurse practitioner and a research officer employed by the aged care organization using predefined eligibility criteria. While this pragmatic approach facilitated recruitment in a complex clinical setting, it may have influenced participant selection. The study population was limited to English‐speaking residents, which may restrict generalizability to culturally and linguistically diverse populations.

The study assessed the feasibility of a new model of collaboration, but the findings may not be generalizable to collaboration involving pharmacists and nurse practitioners with different expertise and experience or outside of the residential aged care context. The time spent by the nurse practitioners, RSMs, GPs, and community pharmacists in implementation of the simplification opportunities was not captured. The relatively high implementation rate of simplification recommendations (47%) suggests that the intervention was acceptable to many residents. However, no residents were interviewed as part of the study, and so it is not possible to conclude directly about the acceptability of residents.

Cost‐benefit analysis was not conducted in this pilot and feasibility study; as a result, it is unclear whether the cost of delivering the intervention would be offset by the potential benefits to the residents and the RACF. This study was also not designed to evaluate the impact of this model of care on resident‐ or service‐level quality indicators. The results of this pilot and feasibility study are valuable for informing future controlled clinical trials of nurse practitioner–pharmacist collaboration to simplify complex medication regimens. These future controlled trials will be important to support a cost‐benefit analysis and determine the impact of this model of care on relevant quality indicators.

## 5. Conclusions

Nurse practitioners and pharmacists can successfully collaborate via telehealth to simplify complex medication regimens for residents of RACFs. The new multidisciplinary team‐based model of care as proposed in this study could encourage more structured collaboration between nurse practitioners, pharmacists, and GPs. This is a promising model for facilitating greater pharmacist involvement in RACFs that do not currently employ an on‐site pharmacist.

## Author Contributions

J. Simon Bell and Angelita Martini conceived the study, obtained grant funding, and participated in the study design. Choon Ean Ooi, Rebecca Walton, and Brigid McInerney were involved in the implementation of the study. Lakkhina Troeung and Choon Ean Ooi conducted the data analyses. Choon Ean Ooi and J. Simon Bell drafted the manuscript.

## Funding

This research study was supported by the Western Australian Nurses Memorial Charitable Trust [Reference Code: WANMCT‐Martini_CRAIK‐01092020].

Open access publishing was facilitated by Monash University, as part of the Wiley–Monash University agreement via the Council of Australasian University Librarians.

## Disclosure

All authors reviewed and approved the final draft of the manuscript.

## Conflicts of Interest

J. Simon Bell is supported by the National Health and Medical Research Council (NHMRC) Boosting Dementia Research Leadership Fellowship and has received grant funding or consulting funds from the NHMRC, Victorian Government Department of Health and Human Services, Dementia Australia Research Foundation, Yulgilbar Foundation, Aged Care Quality and Safety Commission, Dementia Centre for Research Collaboration, Pharmaceutical Society of Australia, Society of Hospital Pharmacists of Australia, GlaxoSmithKline Supported Studies Programme, Amgen, and several aged care provider organizations unrelated to this work. All grants and consulting funds were paid to the employing institution. The other authors declare no conflicts of interest.

## Data Availability

The raw data of this study included sensitive information of the participating residents and hence the data are not shared.
